# The Neuroprotective Effects of Intramuscular Insulin-Like Growth Factor-I Treatment in Brain Ischemic Rats

**DOI:** 10.1371/journal.pone.0064015

**Published:** 2013-05-22

**Authors:** Heng-Chih Chang, Yea-Ru Yang, Paulus S. Wang, Chia-Hua Kuo, Ray-Yau Wang

**Affiliations:** 1 Department of Physical Therapy and Assistive Technology, National Yang-Ming University, Taiwan; 2 Department and Institute of Physiology, National Yang-Ming University, Taiwan; 3 Graduate Institute of Basic Medical Science, Ph.D. Program for Aging, College of Medicine, China Medical University, Taiwan; 4 Department of Biotechnology, College of Health Science, Asia University, Taiwan; 5 Graduate Institute of Exercise Science, Taipei Physical Education College, Taiwan; University of Jaén, Spain

## Abstract

Brain ischemia leads to muscle inactivity-induced atrophy and may exacerbate motor function deficits. Intramuscular insulin-like growth factor I (IGF-I) injection has been shown to alleviate the brain ischemia-induced muscle atrophy and thus improve the motor function. Motor function is normally gauged by the integrity and coordination of the central nervous system and peripheral muscles. Whether brain ischemic regions are adaptively changed by the intramuscular IGF-I injection is not well understood. In this study, the effect of intramuscular IGF-I injection was examined on the central nervous system of brain ischemic rats. Rats were divided into 4 groups: sham control, brain ischemia control, brain ischemia with IGF-I treatment, and brain ischemia with IGF-I plus IGF-I receptor inhibitor treatment. Brain ischemia was induced by right middle cerebral artery occlusion. IGF-I and an IGF-1 receptor inhibitor were injected into the affected calf and anterior tibialis muscles of the treated rats for 4 times. There was an interval of 2 days between each injection. Motor function was examined and measured at the 24 hours and 7 days following a brain ischemia. The affected hind-limb muscles, sciatic nerve, lumbar spinal cord, and motor cortex were collected for examination after euthanizing the rats. IGF-I expression in the central nervous system and affected muscles were significantly decreased after brain ischemia. Intramuscular IGF-I injection increased the IGF-I expression in the affected muscles, sciatic nerve, lumbar spinal cord, and motor cortex. It also increased the p-Akt expression in the affected motor cortex. Furthermore, intramuscular IGF-I injection decreased the neuronal apoptosis and improved the motor function. However, co-administration of the IGF-I receptor inhibitor eliminated these effects. Intramuscular IGF-I injection after brain ischemia attenuated or reversed the decrease of IGF-I in both central and peripheral tissues, and these effects could contribute to neuroprotection and improve motor function.

## Introduction

Motor neuron dysfunction in the brain ischemic regions and in the spinal cord has been noted after brain ischemia. The decrease in the activities of spinal motor neurons may be caused by the adaption after brain ischemia [1]. It has also been suggested that inactivity results in further muscle atrophy in the brain ischemic rats and stroke survivors [2]–[5]. Over time, inactivity-mediated muscle atrophy in the peripheral skeletal muscles after brain ischemia may exacerbate the motor function deficits.

Local injection of insulin-like growth factor-I (IGF-I) into the affected hind-limb muscles has been shown to prevent muscle atrophy after brain ischemia [3]. Furthermore, intramuscular injection of mIGF-I cDNA into the facial muscle of rats after facial nerve injury not only prevents muscle atrophy but also enhances facial nuclei recovery [6]. These results indicate that peripheral intramuscular IGF-I administration may exert the protective effects not just on peripheral skeletal muscles but also on central nervous system (CNS).

IGF-I is found to reduce the extent of ischemic brain injury and promote the exercise-induced protective effects in neurodegenerative animal models [7], [8]. IGF-I activated PI3K/Akt pathway has been suggested to play important roles in regulating cell survival and neuronal plasticity [9], [10]. However, little is known about the changes of IGF-I signaling in the CNS after brain ischemia. In addition, intramuscular IGF-I injection is noted to improve the motor function after brain ischemia [3]. Motor function is suggested to be regulated by the integrity and coordination of the central nervous system and peripheral muscles. Whether the intramuscular IGF-I injection can result in a protective effect of CNS after brain ischemia is not well understood. In this study, we analyzed the changes of IGF-I concentration in the CNS and peripheral skeletal muscles and examined the effects of intramuscular IGF-I injection on brain recovery and motor function performance in the brain ischemic rats.

The results of present study demonstrated that there was decreased IGF-I concentration in the CNS and peripheral skeletal muscles after brain ischemia. This lowering of IGF-I expression may contribute to the observed increases in the neuronal apoptosis and motor function deficits. Intramuscular IGF-I injection within the first week after brain ischemia attenuated or reversed the decrease of IGF-I expression in the CNS and peripheral skeletal muscles, decreased the neuronal apoptosis and improved the motor function. However, such positive effects of intramuscular IGF-I injection were eliminated by the additional application of the IGF-I receptor inhibitor.

## Materials and Methods

### Experimental Animals

Sixty-four adult male Sprague–Dawley rats (body weight = 300–350 g) were used in the present study. All experimental procedures were approved by the Institutional Animal Care and Use Committee of National Yang-Ming University, Taipei, Taiwan (IACUC–941147). After inducting brain ischemia, rats were randomly assigned to the 3 groups: rest control (C), IGF-I treatment (IGF), and IGF-I plus IGF-I receptor inhibitor treatment (IGF+I). A fourth sham ischemic control group (S) was used as surgical control. Each group has two subgroups for examining the protein expression and cortical cell apoptosis (n = 8 for each subgroup). All of the outcome measurements were performed by observers who were blinded to the treatments.

### Brain Ischemia

Surgeries to induce focal ischemia were conducted under pentobarbital anesthesia (50 mg/kg, inducing anesthesia that lasted for a minimum of 2 hours). The rats underwent right middle cerebral artery occlusion (MCAO) to induce brain ischemia in accordance with previously described procedures [11], [12]. During surgery, the rectal temperature of rats was monitored and maintained at 37.0°C ±0.5°C using a heating blanket with an electronic temperature controller (WATLOW 050100C1, Bowdoinham, USA). After suturing and recovery, the rats were returned to their cages. A neurological grading system described by Menzies et al., was used to perform neurological examinations at the 24 hours post-MCAO [13]. Rats with the neurological score less than 2 were excluded from the study. The median neurological scores were 2 (range: 2–3), 3 (range: 2–3) and 3 (range: 2–3) for the C, IGF and IGF+I groups, respectively. There were no significant differences in the neurological score among the brain ischemic groups.

### IGF-I and IGF-I Receptor Inhibitor Injection

Rats in the IGF and IGF+I groups received intramuscular IGF-I injections (200 ng/100 µL). IGF-I was injected locally into the left calf and anterior tibialis (TA) muscles under light ether anesthesia at the 24 hours post-MCAO and on every other day, for total 4 injections within 7 days post-MCAO. Rats in the S and C groups received intramuscular injections of 0.1 M PBS (100 µL, pH 7.2) in the left calf and TA muscles at comparable times. Rats in the IGF+I group also received an additional intramuscular injection of IGF-I receptor inhibitor (AG1024, 30 µg/100 µL) 30-min prior to receiving each IGF-I injection. The IGF-I powder (Sigma, USA) was dissolved in 0.1 M PBS. The AG1024 powder (3-bromo-5-t-butyl-4-hydroxy-benzylidene- malonitrile, Invitrogen, USA) was first dissolved in DMSO and then diluted with 0.1 M PBS.

### Motor Function Measurement

The parallel bar crossing test was used to test motor function [14]. In each trial, rats were placed on a platform and encouraged to walk on the parallel bars for 1 min. Instances in which their hind paws were placed on one bar, when a hind paw slipped over the bar, or when they fell or swung from the bars were recorded as errors. The errors that were made per meter were calculated. In order to determine the effect of IGF-1 treatment on the motor function improvement, this motor function test was administered at the 24 hours post-MCAO and 7 days post-MCAO.

### Sample Preparation

Eight rats from each group were anesthetized 1 hour after the final motor function test. The portions of left sciatic nerve (thigh segment), total lumbar spinal cord [15], and right motor cortex [16] were removed, weighed, washed with cold saline twice and homogenized in the buffer A, which contained 50 mmol/L Tris-Cl (pH 8.0), 150 mmol/L NaCl, 0.02% sodium azide, 0.1% SDS, 1 µg/mL aprotinin, 1% Nonidet P-40, 0.5% sodium deoxycholate, and 0.2 mmol/L phenylmethylsulfonyl fluoride (PMSF). The gastrocnemius, soleus (S), and TA muscles in the left side were carefully removed, weighed and washed with cold saline twice. The red gastrocnemius (rG) and white gastrocnemius (wG) were separated. A 0.5-cm thickness of the muscle belly cut from the midline of each muscle was used as the muscle sample. The muscle samples were homogenized in the buffer B, which contained 0.02 mol/L HEPES, 0.25 mol/L sucrose, 0.2% sodium azide, and 0.2 mmol/L PMSF. The lysis buffers also included a protease inhibitor cocktail (1∶100). The lysates were centrifuged at 12,500×*g* for 30 min at 4°C, and the supernatants were collected and stored at −80°C. The plasma sample was collected from 1 ml blood with heparin (10 I.U.) by heart puncture, centrifuged at 10,000×*g* for 1 min and stored at −20°C.

### IGF-I Measurement

Quantikine mouse IGF-I ELISA kits (R&D Systems, USA) were used in accordance with the manufacturer’s instructions to measure IGF-I concentrations in the tissue and plasma samples. The plasma and tissue supernatants from the affected hind-limb muscles, sciatic nerve, lumbar spinal cord, and motor cortex were diluted 1000-fold and 6-fold, respectively, with calibrator diluent RD5-38 buffer. The calibrator diluent buffer (50 µL) was added to each well, followed by the addition of 50 µL of sample, including standard dilutions. The plates were incubated at room temperature on a horizontal orbital microplate shaker (500±50 rpm) for 2 hours. After aspirating the unbound samples and washing each well, 100 µL mouse IGF-I conjugate was added and incubated at room temperature and then shaking for 2 hours. After that, 100 µL of substrate solution was added and incubated at room temperature in the dark for 30 min. Finally, 100 µL of stop solution was added, and the plate was gently tapped to ensure mixing. The optical density of each well was determined with a microplate reader at 450 nm. The IGF-I concentration in each sample was calculated from the standard curve and presented as ng/g for tissues and ng/mL for plasma.

### Cortical Phosphorylated-Akt Measurement

The relative expressions of PI3/Akt signal in the affected motor cortices were determined by western blotting. The total protein of cortical supernatant was measured using Bradford-red protein assay. After adding the sample buffer containing 0.1 M Tris-Cl (pH 6.8), 25% glycerol, 2% SDS, 0.02% bromophenol blue, and 5% β-mercaptoehtanol, the sample was boiled 10 min. Total 30 µg protein of each sample was resolved in 8% SDS-polyacrylamide gel and then transferred onto polyvinylidene fluoride membrane (Millipore Corp., USA). The membrane was blocked with 0.1% Tween 20/Tris buffered saline (TBST) containing 5% nonfat milk at room temperature for 1 hour following probed with the primary rabbit-anti phosphorylated-Akt^ser473^ antibody (p-Akt, 1∶1000, Millipore, USA) at 4°C overnight. After washing three times with TBST, the membrane was incubated with horseradish peroxidase-conjugated goat anti-rabbit IgG secondary antibody (1∶6000, Chemicon, USA) at room temperature for 1 hour. After washing three times with TBST, the resulting signals were visualized with a chemiluminescence reagent (ECL Plus; Amersham Biosciences, UK). After stripping, washing and another blocking, the membrane was re-probed with rabbit-anti total-Akt (t-Akt) primary antibody (1∶1000, Millipore, USA) at 4°C overnight. The procedures for determining the signals of t-Akt were the same as described above. The relative protein expression was quantified by densitometry using image analysis software (ImageQuant; Amersham Biosciences, UK). The ratio of p-Akt to t-Akt was calculated and presented as a percentage to that of the S group.

### Cortical Cell Apoptosis Measurement

An additional eight rats in each group were fixed on a stereotaxic frame under anesthesia, and two burr holes were drilled at AP: +2.52 mm; R: +3 mm and AP: +1.56 mm; R: +3 mm from the bregma [16]. A micro-injection needle was stabbed lightly into the cortical surface. The rats were euthanized by intra-cardiac perfusion with 0.1 M PBS followed by 4% paraformaldehyde (pH 7.2). The brain was removed, and coronal sections (20 µm per section) through the needle marks as described above were cut with a cryostat and affixed to slides. The cortical apoptotic cells were measured using an *in situ* cell death detection kit (Roche, USA) according to the manufacturer instructions. In brief, the slides were firstly heated at 60°C for 30 min and incubated with proteinase K (10 µg/mL in 10 mM Tris-HCl, pH 7.4) at room temperature for 30 min. After incubating with 0.1% sodium citrate containing 0.1% Triton X-100 for 20 min on ice, the terminal transferase dUTP nick end-labeling (TUNEL) solution was added to each section, and the slides were incubated in the dark at 37°C for 60 min. The slides were then incubated with Hoechst 33258 (4 µg/mL) in the dark for 10 min at room temperature. The slides were rinsed with 0.1 M PBS twice between each of the aforementioned steps. To quantify apoptotic cells and total cells, the numbers of TUNEL-labeled and Hoechst-positive cells in the peri-infarct area were counted. The ratio of TUNEL-labeled cells to Hoechst-positive cells was calculated in each group.

### Statistical Analysis

The results of cortical cell apoptosis, IGF-I concentration, p-Akt expression and motor function were presented as the mean ± standard error of means. All variables were first analyzed by the Kolmogorov-Smirnov test to confirm the normality of variables. Results of the cortical cell apoptosis, IGF-I concentration and p-Akt expression were examined by one-way analysis of variance (ANOVA) with a post-*hoc* Tukey test. The results of motor function were analyzed by repeated two-way ANOVA. The correlation between IGF-I concentration and motor function deficit (7 days post-MCAO) was examined by Pearson correlation. The significance level was set at *P*<0.05.

## Results

### IGF-I Concentrations

**Figure 1:**
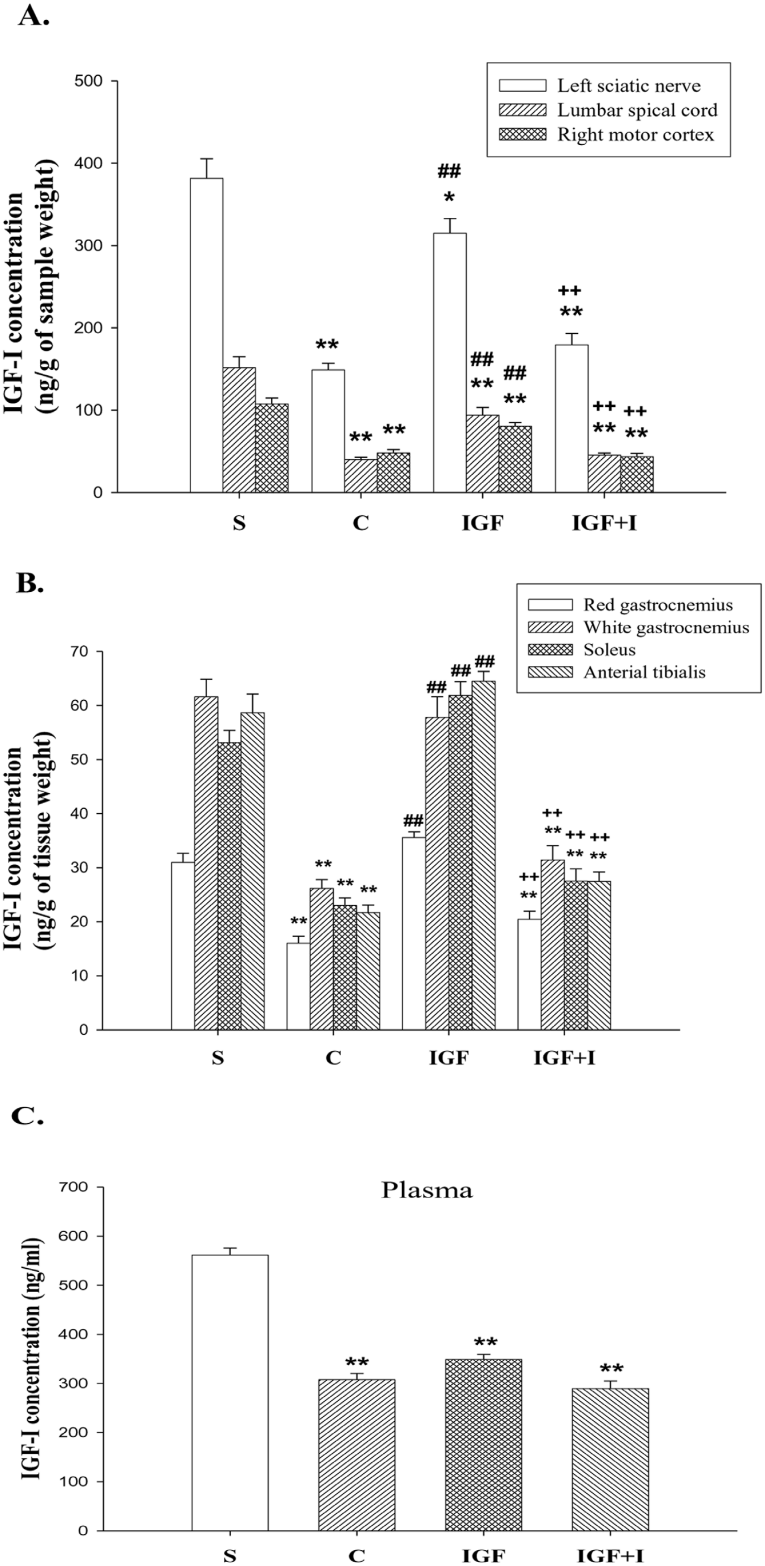
The IGF-I concentrations in the tissue samples and plasma (n = 8 for each group). **A:** The IGF-I concentrations of the left sciatic nerve, total lumbar spinal cord and right motor cortex. **B:** The IGF-I concentrations of the left hind-limb muscles. **C:** The IGF-I concentrations of the plasma. *, **, *P*<0.05, *P*<0.01 vs. S group; ##, *P*<0.01 vs. C group; ++, *P*<0.01 vs. IGF group. S: Sham control; C: MCAO control; IGF: MCAO with IGF-I treatment; IGF+I: MCAO with AG1024 and IGF-I treatment.

The Kolmogorov-Smirnov test confirmed the normality of this variable among groups (*P*>0.05). The IGF-I concentrations of the left sciatic nerve, total lumbar spinal cord, and right motor cortex in the C group were significantly decreased when compared with those of the S group (sciatic nerve, *P*<0.01; lumbar spinal cord, *P*<0.01; motor cortex, *P*<0.01) (Figure 1A). The IGF-I concentrations of these tissues in the IGF group were significantly higher than those in the C group (sciatic nerve, *P*<0.01; lumbar spinal cord, *P*<0.01; motor cortex, *P*<0.01), although there were remained significantly lower than those in the S group (sciatic nerve, *P*<0.05; lumbar spinal cord, *P*<0.01; motor cortex, *P*<0.01). Moreover, the effect of intramuscular IGF-I injection on the expression of IGF-I in the CNS was eliminated by the IGF-I receptor inhibitor, as demonstrated by the IGF+I group (sciatic nerve, *P*<0.01; lumbar spinal cord, *P*<0.01; motor cortex, *P*<0.01).

The IGF-I concentrations of the affected hind-limb muscles in the C group were significantly decreased when compared with those of the S group (rG, *P*<0.01; wG, *P*<0.01; S, *P*<0.01; TA, *P*<0.01) (Figure 1B). However, the IGF-I concentrations of these muscles in the IGF group were significantly higher than those in the C group (rG, *P*<0.01; wG, *P*<0.01; S, *P*<0.01; TA, *P*<0.01) and did not significantly differ from those in the S group. However, these high IGF-I concentrations resulted by the IGF group were eliminated by the IGF-I receptor inhibitor, as demonstrated by the IGF+I group (rG, *P*<0.01; wG, *P*<0.01; S, *P*<0.01; TA, *P*<0.01).

The plasma IGF-I concentration in the C group (*P*<0.01), IGF group (*P*<0.01), and the IGF+I group (*P*<0.01) were all significantly lower than that in the S group (Figure 1C). There were no significant differences in the plasma IGF-I concentration among the C, IGF and IGF+I groups (*P*>0.05).

### Cortical Cell Apoptosis

**Figure 2:**
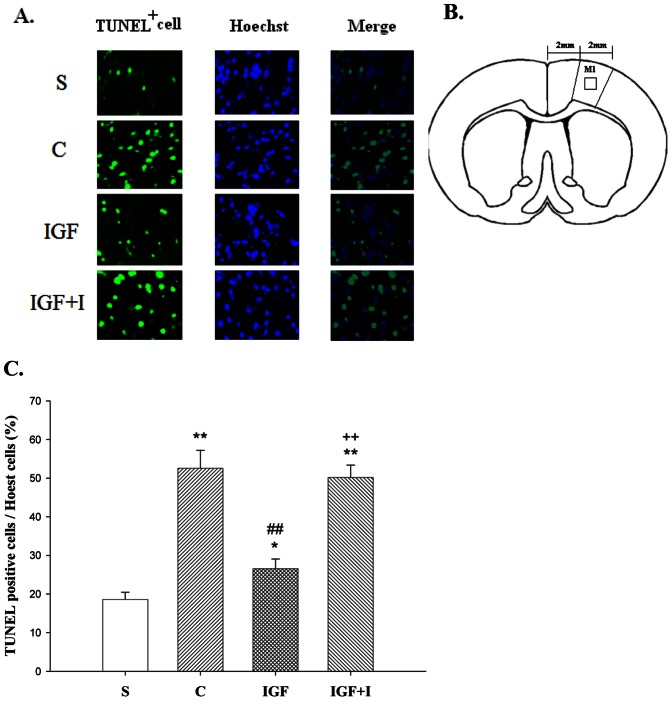
The relative expression of apoptotic cells in the peri-infarct area (primary motor cortex, M1) (n = 8 for each group). **A:** Expression of TUNEL positive and Hoechst cells among groups. **B:** The peri-infarct area for measurement. **C:** Quantification of the percentage of TUNEL positive cells to Hoechst cells. *, **, *P*<0.05, *P*<0.01 vs. S group; ##, *P*<0.01 vs. C group; ++, *P*<0.01 vs. IGF group. S: Sham control; C: MCAO control; IGF: MCAO with IGF-I treatment; IGF+I: MCAO with AG1024 and IGF-I treatment.

The Kolmogorov-Smirnov test confirmed the normality of this variable among groups (*P*>0.05). The expressions of TUNEL-labeled and Hoechst-positive cells among groups are shown in the Figure 2A. The illustration of the peri-infarct area for measurement is presented in the Figure 2B. The ratio of TUNEL-labeled cells to Hoechst-positive cells in the C group was significantly increased when compared with that of the S group (*P*<0.01) (Figure 2C). The ratio of TUNEL-labeled cells to Hoechst-positive cells in the IGF group was significantly lower than that in the C group (*P*<0.01) but remained higher than that in the S group (*P*<0.05). The protective effect of intramuscular IGF-I injections against cortical cell apoptosis was eliminated by the IGF-I receptor inhibitor, as demonstrated by the IGF+I group (*P*<0.01).

### Cortical Phosphorylated-Akt Expression

**Figure 3:**
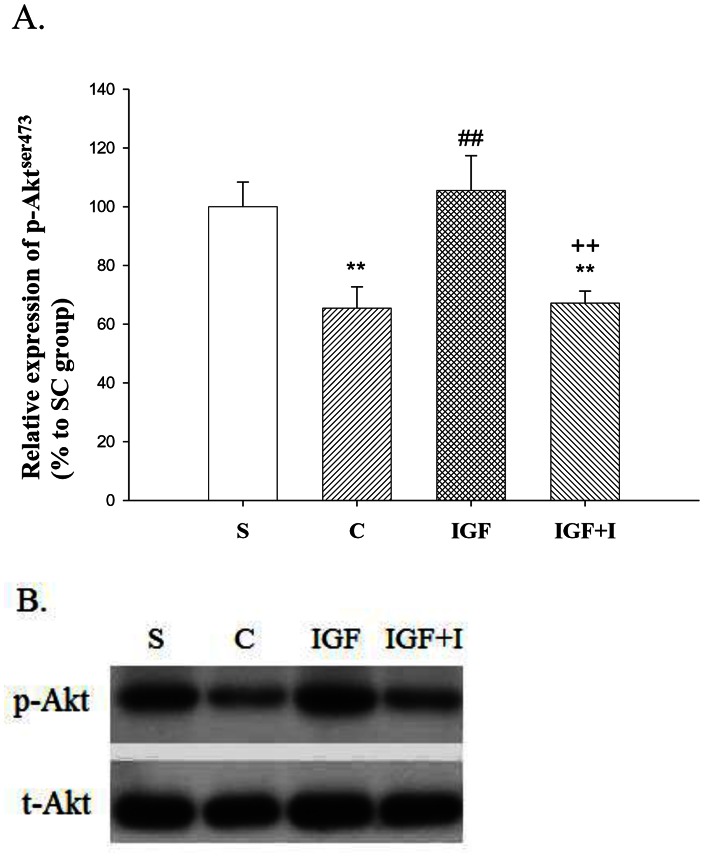
The relative expression of p-Akt in the right motor cortex (n = 8 for each group). **A:** The quantification of the relative expression of p-Akt. All of the data are represented as the ratio of p-Akt band density to t-Akt band density and presented as a percentage to the p-Akt to t-Akt ratio of the S group. **B:** Western blot results. **, *P*<0.01 vs. S group; ##, *P*<0.01 vs. C group; ++, *P*<0.01 vs. IGF group. S: Sham control; C: MCAO control; IGF: MCAO with IGF-I treatment; IGF+I: MCAO with AG1024 and IGF-I treatment.

The Kolmogorov-Smirnov test confirmed the normality of this variable among groups (*P*>0.05). The expression of p-Akt in the C group was significantly decreased when compared with that of the S group (*P*<0.01) (Figure 3). The expression of p-Akt in the IGF group was significantly higher than that in the C group (*P*<0.01), and did not demonstrate significant difference with that in the S group (*P*>0.05). However, the enhancement of p-Akt level by intramuscular IGF-I injection was eliminated by the IGF-I receptor inhibitor, as demonstrated by the IGF+I group (*P*<0.01).

### Motor Function Performance

**Figure 4:**
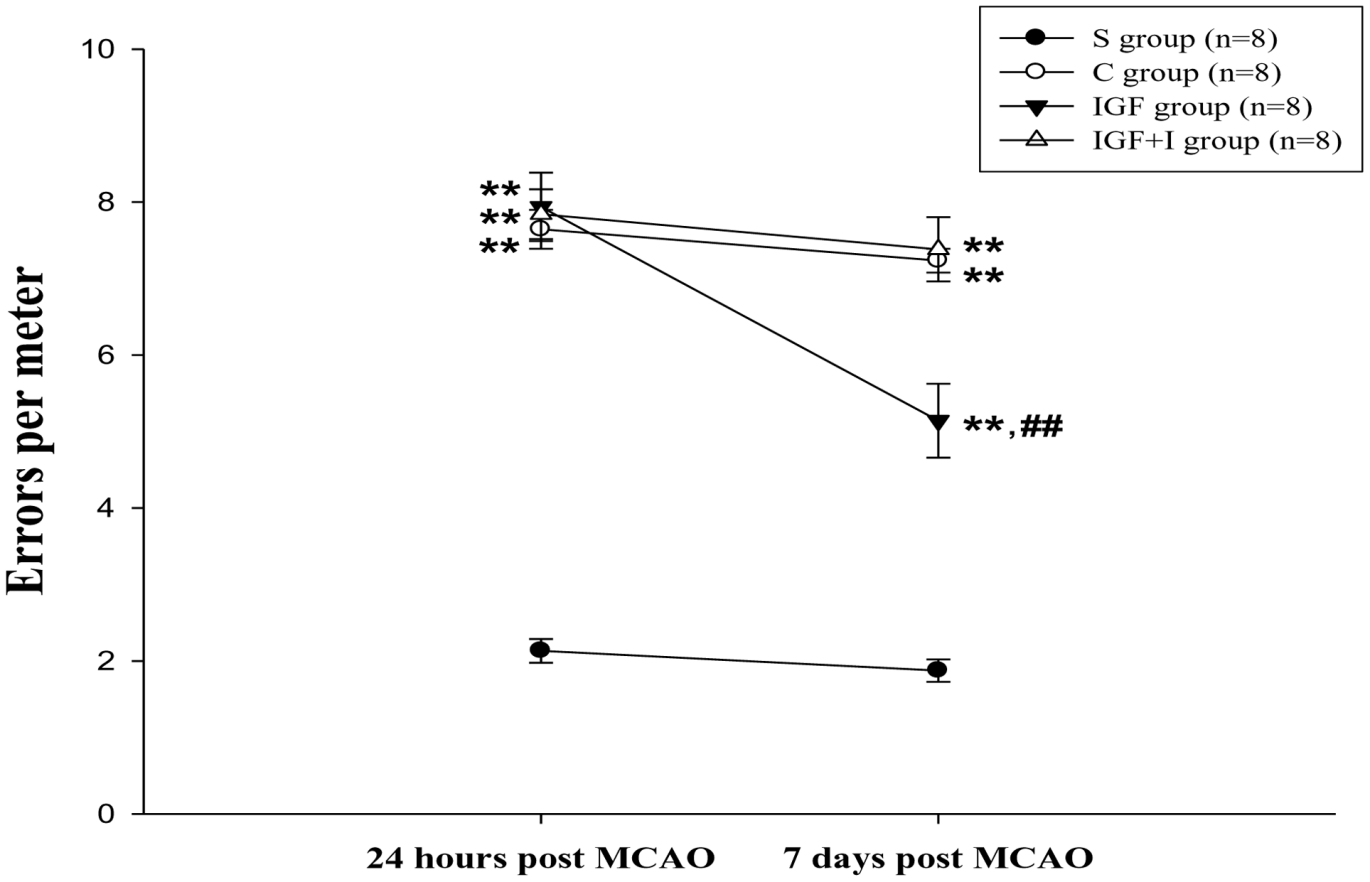
The motor performances in the parallel bar crossing test (n = 8 for each group). **, *P*<0.01 vs. S group; ##, *P*<0.01 vs. C group. S: Sham control; C: MCAO control; IGF: MCAO with IGF-I treatment; IGF+I: MCAO with AG1024 and IGF-I treatment.

The Kolmogorov-Smirnov test confirmed the normality of this variable among brain ischemic groups (*P*>0.05). Significant motor function impairment was noted in the brain ischemic rats when compared with the S group in both measurement time point (*P*<0.01) (Figure 4). However, the motor function of the IGF group measured at the 7 days post-MCAO was significantly improved when compared with that of the C group (*P*<0.01). The motor performance of the IGF+I group did not significantly differ from that in the C group (*P*>0.05). Furthermore, motor function deficit was negatively correlated with the IGF-I concentration in the affected motor cortex (r = −0.806, *P*<0.01), lumbar spinal cord (r = −0.890, *P*<0.01), and skeletal muscles (rG: r = −0.728, *P*<0.01; wG: r = −0.798, *P*<0.01; S: r = −0.772, *P*<0.01; TA: r = −0.801, *P*<0.01).

## Discussion

Recent articles have demonstrated that general exercise improves healthy rat brain functions and fasten the recovery of ischemic rat brain functions by enhancing IGF-I signaling in rat brains [8], [17], [18]. IGF-I-mediated PI3K/Akt pathway has also been suggested to play important roles in inhibiting cell apoptosis and promoting cell metabolism in both neuronal cells and peripheral muscle cells [9], [10], [19]. IGF-I is also shown to be able to modulate the neuronal activity [11], [20]. In this study, the IGF-I concentrations were all significantly decreased after a brain ischemia for rats, in the affected motor cortex, lumbar spinal cord, sciatic nerve, and hind-limb muscles. The decrease of IGF-I concentration was ameliorated by the intramuscular IGF-I injection. The study showed the motor function was negatively correlated with the IGF-I concentration of the affected motor cortex (r = −0.806), lumbar spinal cord (r = −0.890), and peripheral muscles (rG: r = −0.728; wG: r = −0.798; S: r = −0.772; TA: r = −0.801). These results imply that IGF-I signaling of central neuronal cell and peripheral muscle is a critical important contributing factor on motor function recovery in brain ischemic rats.

Inhibition of IGF-I signal transduction in the affected skeletal muscle has been noted to lessen the ameliorating effect of IGF-I on muscle atrophy after brain ischemia [3]. In the present study, we noted the neuronal apoptosis was significantly decreased after intramuscular IGF-I treatment. Also, an inhibition of IGF-I signaling in the affected skeletal muscle negates the effect of intramuscular IGF-I injection on neuronal cell apoptosis. These results suggest that IGF-I signaling in the peripheral skeletal muscle plays important roles not just in modulating peripheral muscle metabolism but also in regulating brain recovery after brain ischemia. Such protective effect is shown to be related to the attenuation or the reversion of the decrease in IGF-I concentration in central neuronal cells of rats.

In the present study, the p-Akt expression in the affected skeletal muscles of brain ischemic rats with intramuscular IGF-I injection was significantly increased when compared with that of the brain ischemia control rats [3]. The muscular IGF-I concentration was significantly increased in rats with IGF-I injection but not in rats with both IGF-I plus IGF-I receptor inhibitor injections. This result shows that the increases of IGF-I concentration in these muscle samples are due to the IGF-I synthesis in the injected muscles, not by the direct effects of the exogenous injected IGF-I. The IGF-I produced by the muscles may then participate in preventing brain ischemia-induced muscle atrophy and thereby contribute to motor function improvement.

The interesting finding of this study is that intramuscular IGF-I injection exerts neuroprotective effects for brain ischemic rats. As such, it can be assumed that the presence of a retrograde, or upstream, flow of information to the CNS, may possess feedback functions [21]. However, the molecular details of the signaling system between periphery and CNS are still not fully understood. Muscle itself is a source of neurotrophic and growth factors that may have protective effect on motor neurons [22]. Previous studies have shown that the over-expression of IGF-I in muscle attenuates motor neuron loss in animal models [23], [24]. It has been suggested that IGF-I is bilaterally transported in the sciatic nerve and facilitates neuronal sprouting on skeletal muscles in animal models [25]–[27]. Moreover, IGF-I is able to cross the blood-brain barrier [28]. Previous studies indicated that systemic administration of IGF-1 by either intravenous or subcutaneous injection results in a decreased infarct volume [9], [29]. Therefore, the blood circulation and/or nerve retrogression-mediated IGF-I transport may contribute the cross-talk between the CNS and peripheral skeletal muscles. However, we found the IGF-I concentration of plasma was not changed after intramuscular IGF-I injections. Previous studies also indicate that neither the over-expression nor the exogenous administration of IGF-I in the muscle alter serum IGF-I concentration [30]–[32]. Therefore, intramuscular IGF-I injection may reverse the decrease of IGF-I concentration in the CNS partly through the nerve retrogression system.

Muscle-released brain-derived neurotrophic factor (BDNF) has also been suggested to be transported in a retrograde manner by motor neurons [33]. The BDNF is also suggested to be a survival-promoting factor which protects neurons in vitro and in vivo [34]. The increase or maintenance of IGF-I expression in the affected skeletal muscles may also increase the muscle-released trophic factors, thus provide additive protective effects. A lessened decrease in cell damage after ischemia may in turn contribute to more neurotrophic factors expression in the CNS and therefore contribute to improvement of motor performance. However, the major challenges for retrograde transport are the distance and gaps between the peripheral skeletal muscles and the CNS.

In this study, the prevention of muscle atrophy after brain ischemia may also maintain or increase the utilization of skeletal muscles, thereby attenuating the adaptive degenerations of the CNS and increasing the cross-interactions between the skeletal muscles and the CNS. This is the first study to demonstrate that peripheral intramuscular IGF-I application can result in brain recovery after brain ischemia. Further studies are needed to investigate the possible underlying mechanisms.

### Conclusion

This study indicates that decreases of IGF-I concentration in both cortical cells and peripheral skeletal muscles of rats may contribute to motor function impairment after brain ischemia. Intramuscular IGF-I injection attenuates or reverses the decrease of IGF-I signaling in both cortical cells and skeletal muscles and thus reduces the cortical cell apoptosis induced by brain ischemia. Also, the prevention of cortical cell death by intramuscular IGF-I treatment is therefore contributed to the motor function improvement after brain ischemia.
